# Supplementary material for: Tuning reactivity of Fischer–Tropsch synthesis by regulating TiOx overlayer over Ru/TiO2 nanocatalysts
Source: Nat Commun. 2020 Jun 24;11:3185. doi: 10.1038/s41467-020-17044-4 (PMC7314765; doi:10.1038/s41467-020-17044-4)
Supplement: Supplementary file 1 — Supplementary Information [file 41467_2020_17044_MOESM1_ESM.pdf]

Supplementary Information

Tuning reactivity of Fischer–Tropsch synthesis by regulating TiO_x overlayer over Ru/TiO_2 nanocatalysts

Zhang et al.

Supplementary Information

Tuning reactivity of Fischer–Tropsch synthesis by regulating TiO_x overlayer over Ru/TiO_2 nanocatalysts

Yaru Zhang[1,2], Xiaoli Yang[1,2,4], Xiaofeng Yang[1,*], Hongmin Duan[1], Haifeng Qi[1,2], Yang Su[1], Binglian Liang[1], Huabing Tao[3], Bin Liu[3], De Chen[4], Xiong Su[1,*], Yanqiang Huang[1,*], and Tao Zhang[1]

[1] State Key Laboratory of Catalysis, Dalian Institute of Chemical Physics, Chinese Academy of Sciences, Dalian 116023, China

[2] University of Chinese Academy of Sciences, Beijing 100049, China

[3] School of Chemical and Biomedical Engineering, Nanyang Technological University Singapore 637459, Singapore

[4] Department of Chemical Engineering, Norwegian University of Science and Technology, Trondheim 7494, Norway.

*To whom correspondence should be addressed. E-mail: yqhuang@dicp.ac.cn (Y. Huang); yangxf2003@dicp.ac.cn (X. Yang); suxiong@dicp.ac.cn (X. Su).

Supplementary Methods

Preparation of the Ru/Al_2O_3-450 catalyst. The Ru/Al_2O_3-450 catalyst was prepared by an incipient wetness impregnation method. In a typical synthesis, 1.6 g of the aqueous $RuCl_3 \cdot 3H_2O$ solution was diluted with deionized water to give a homogenous Ru solution, with a near saturated water absorption capacity of the support. Subsequently, 2.0 g of Al_2O_3 was added to the solution, followed by the ultrasonication for 30 min to ensure dispersion of the oxide. The suspension was allowed to stand overnight, followed by drying at 120 °C for 12 h. The resulting material, denoted as Ru/Al_2O_3-450, was reduced in a H_2 gas flow (20 mL min^{-1}) at 450 °C before catalytic performance tests.

XRD. Powder X-ray diffraction (XRD) data were acquired using a PANalytical X'Pert-Pro X-ray diffractometer with Cu Kα radiation (λ = 0.1541 nm), operated at 40 kV and 40 mA. A continuous-scan mode was used to collect 2θ data from 10° to 80° at a scan rate of 10° min^{-1}.

N_2 physisorption. Nitrogen physisorption was performed with a Micromeritics ASAP 2460 instrument at −196 °C. The samples were degassed in vacuum at 110 °C for 1 h, then 300 °C for 4 h before measurements. The specific surface areas (S_{BET}) were calculated using the Brunauer-Emmett-Teller (BET) method over the relative pressure range of P/P_0 = 0.05–0.30. The pore volumes (V_{pore}) were determined using the single point adsorption total pore volume of pores less than 40.3 nm in diameter at P/P_0 = 0.95.

HRSEM. The morphology of each catalyst was characterized by high-resolution

scanning electron microscopy (HRSEM) using a field-emission JEOL JSM-7800F microscope operating at 3.0 kV.

H_2-TPR experiment. H_2 temperature programmed reduction (H_2-TPR) was performed with a Micromeritics AutoChem II 2920 apparatus. Prior to TPR measurements, the pristine Ru/TiO_2 sample was loaded into a quartz reactor and pretreated with Ar at 200 °C for 60 min. After the temperature decreasing to 50 °C, a 10% H_2 in Ar flow was introduced into the reactor by heating the sample from 50 to 800 °C at a heating rate of 10 °C min^{-1}. The signal was recorded online with a thermal conductivity detector (TCD).

Cu upd experiments. The underpotential deposition of Cu (Cu upd) was employed to determine the exposure of surface metallic Ru on the Ru/TiO_2-x catalysts. Copper is an ideal metal for upd on Ru because of the similarity of the atomic radii of the two metals—Cu, 0.128 nm; Ru, 0.134 nm. The specific surface area of Ru (i.e. S_{sp} of Ru, m^2 g^{-1}) can be calculated by the integration of the peak area corresponding to upd stripping. In this process, we assume that a single Cu atom deposits on one surface Ru to form a monolayer deposition, which can be realized by judicious choice of electrochemical potential and deposition time.

$$Cu_{upd} \rightarrow Cu^{2+} + 2e^{-} \quad 420\ \mu C\ cm^{-2} \qquad (1)$$

It is worth noting that only the Ru species in reduction state can act as deposition site for Cu upd. In contrast, the Ru species in oxidation state are inert site for Cu upd. Therefore, only the amount of surface metallic Ru can be acquired from Cu upd experiments, which in turn can provide the residual metallic Ru sites after covering by

TiO_x overlayers. These analyses were carried out in a solution containing 0.1 mol L^{-1} H_2SO_4 and 0.002 mol L^{-1} $CuSO_4$. Electrodes were cleaned electrochemically then transferred into the solution containing dissolved cupric ions, after which they were polarized at 0.3 V for 100 s. A linear voltammetry scan was then performed from the admission potential to the point at which all the underpotential deposition Cu was oxidized, at a scan rate of 0.01 V s^{-1}. The specific surface area of the metallic Ru was calculated using the equation

$$S_{sp} = \frac{Area\ of\ surface\ metallic\ Ru}{Mass\ of\ catalyst \times \omega_{Ru}} \quad (2)$$

where the numerator was determined from the Cu upd data and ω_{Ru}, the mass fraction of Ru, was determined by ICP-OES.

The dispersion of the metallic Ru, D, was determined by the equation

$$D = S_{sp} \times \frac{M_{Ru}}{N_A \times a_m} \quad (3)$$

where M_{Ru} is the atomic mass of Ru (101.07 g mol^{-1}), N_A is Avogadro's number (6.02 × 10^{23} mol^{-1}) and a_m is the area occupied by a surface atom (for Ru, a_m = 6.35 Å^2).

XPS measurements. X-ray photoelectron spectroscopy (XPS) data were obtained using a Thermofisher ESCALAB 250Xi instrument, employing monochromated Al Kα radiation ($h\upsilon$ = 1486.6 eV) as the X-ray source. The samples were pretreated at different temperatures in a H_2 flow and then held under an inert atmosphere, followed by rapid transfer to the sample chamber to minimize exposure to air. The results were calibrated by setting the C 1*s* adventitious carbon peak position to 284.6 eV.

The calculation method for FTS catalytic performance. The feed gas (H_2/CO/Ar = 64/32/4) and the gaseous products (including CO_2, CH_4 and C_2–C_4 hydrocarbons)

were analyzed online by gas chromatograph (GC), in which Ar was used as an internal standard to calculate the extent of CO conversion and product selectivity. The catalytic results were determined by the peak areas of the components identified by GC which was equipped with an HP-PLOT/Q capillary column connected to a flame ionization detector (FID) and a TDX-01 column connected to a thermal conductivity detector (TCD).

The thermal conductivity detector (TCD) was used to detect inorganic gaseous, including Ar, CO, CH_4 and CO_2. The CO conversion, CH_4 selectivity and CO_2 selectivity can be determined by the peak areas of the components identified by TCD.

The CO conversion, X_{CO}, was calculated using the equation

$$X_{CO} = \frac{n_{in}(CO) - n_{out}(CO)}{n_{in}(CO)} = 1 - \frac{A_{out}(CO)/A_{out}(Ar)}{A_{in}(CO)/A_{in}(Ar)} \quad (4)$$

where $n_{in}(CO)$ and $n_{out}(CO)$ refer to the mole number of CO at the inlet and outlet, respectively, $A_{in}(CO)$ and $A_{in}(Ar)$ refer to the chromatographic peak area of CO and Ar in the feed gas, and $A_{out}(CO)$ and $A_{out}(Ar)$ refer to the chromatographic peak area of CO and Ar in the off-gas.

The reaction rate was calculated as

$$\text{Reaction rate} = \frac{GHSV \times X_{CO} \times CO\ concentration}{22400 \times \omega_{Ru}} \quad (5)$$

where $GHSV$ is the gas hourly space velocity and ω_{Ru} is the mass fraction of Ru (2.2 wt% detected by ICP-OES).

The turnover frequency (TOF) was determined using the equation

$$\text{TOF} = \frac{Reaction\ rate \times M_{Ru}}{3600 \times Ru\ dispersion} \quad (6)$$

where M_{Ru} is the atomic mass of Ru (101.07 g mol^{-1}) and the Ru dispersion was

determined by the CO chemisorption results.

The selectivity values presented in this work were calculated on a carbon basis.

The selectivity of CO_2 was calculated as

$$S_{CO_2} = \frac{n_{out}(CO_2)}{n_{in}(CO)-n_{out}(CO)} = \frac{f_{CO_2/Ar}[A_{out}(CO_2)/A_{out}(Ar)]}{f_{CO/Ar}[A_{in}(CO)/A_{in}(Ar)-A_{out}(CO)/A_{out}(Ar)]} \quad (7)$$

where $f_{CO2/Ar}$ is the relative correction factors of CO_2 to Ar, which was determined by the calibrating gas; $A_{out}(CO_2)$ refers to the chromatographic peak area of CO_2 detected by TCD in the off-gas.

Similarly, the selectivity of CH_4 was calculated as

$$S_{CH_4} = \frac{n_{out}(CH_4)}{n_{in}(CO)-n_{out}(CO)} = \frac{f_{CH_4/Ar}[A_{out}(CH_4)/A_{out}(Ar)]}{f_{CO/Ar}[A_{in}(CO)/A_{in}(Ar)-A_{out}(CO)/A_{out}(Ar)]} \quad (8)$$

where $f_{CH4/Ar}$ is the relative correction factors of CH_4 to Ar, which was determined by the calibrating gas; $A_{out}(CH_4)$ refers to the chromatographic peak area of CH_4 detected by TCD in the off-gas.

The flame ionization detector (FID) were used to detect CH_4 and C_2–C_4 hydrocarbons. The CH_4 selectivity was used as a bridge to calculate the selectivity of C_2–C_4 hydrocarbons identified by FID.

The selectivity for C_xH_y (x = 2–4) hydrocarbons was calculated as

$$S_{C_xH_y} = \frac{x \cdot n_{out}(C_xH_y)}{n_{in}(CO)-n_{out}(CO)} = x \cdot f_{C_xH_y/CH_4} \cdot \frac{A_{FID}(C_xH_y)}{A_{FID}(CH_4)} \cdot S_{CH_4} \quad (9)$$

where $f_{CxHy/CH4}$ is the relative correction factors of C_xH_y to CH_4, which was determined by the calibrating gas; $A_{FID}(CH_4)$ and $A_{FID}(C_xH_y)$ refer to the chromatographic peak area of CH_4 and C_xH_y detected by FID in the off-gas, S_{CH4} is the CH_4 selectivity calculated by TCD.

Carbon balances were all greater than 90% and the selectivity for C_{5+} was

determined using the relationship

$$S_{C_{5+}} = 100\% - S_{CO_2} - S_{C_1} - S_{C_2} - S_{C_3} - S_{C_4} \quad (10)$$

The carbon number distribution in C_{5+} fraction, including liquid hydrocarbons and solid wax, was analyzed offline using an Agilent 7890 gas chromatograph equipped with an HP-5 capillary column connected to a flame ionization detector (FID). The liquid hydrocarbons were dissolved in ethanol, while the solid wax was dissolved in dodecane. The relative content of each peak was detected by the normalization method of peak area.

DFT calculations. The relative stability of different TiO_x (x = 1–4) clusters on the Ru(001) surface under different reduction degree conditions which can be represented as the variation of chemical potential of oxygen, was calculated according to the procedure of previous research[1].

Considering a successive reduction of TiO_4/Ru(001) to TiO_2/Ru(001), the energy of removing an oxygen (ΔE_r) on TiO_x/Ru(001) can be expressed as

$$\Delta E_r = E(TiO_{x-1}) + \mu_o - E(TiO_x) \quad (11)$$

Here, the chemical potential of O atom (μ_o) is restrained between

$$\mu_{o2} + 1/2\, H_f(TiO_2) < \mu_o < \mu_{o2} \quad (12)$$

$$\mu_{o2} = 1/2\, E(O_2) \quad (13)$$

due to the limitation of the non-condensed condition of Ti metal and O_2 solid on our Ru/TiO_x catalysts, which corresponds to the O-poor and O-rich conditions, respectively. μ_{o2} refers to the chemical potential of gaseous O_2. $E(O_2)$ is the total

energy of a free O_2 molecule, and the data of the formation energy of rutile TiO_2 ($H_f(TiO_2)$) was acquired from the reference (−10.30 eV)[2].

The relativistic DFT calculations were performed using the VASP code (a version of 5.4.4). The Perdew-Burke-Ernzerhof (PBE) exchange-correlation functional was used. The core and valence electrons were represented by the projector augmented wave (PAW) potential, and the plane wave basis set with a cut-off energy of 500 eV was used. The core and valence electrons were represented by the projector augmented wave potential updated in 2012 (potpaw_PBE.5.2), which has been proved to acquire a reliable chemical accuracy in solid calculations[3]. The valence electrons were designated of Ti ($3d^34s^1$), O ($2s^22p^4$), Ru ($4d^75s^1$), and C ($2s^22p^2$) for the initial geometry searching and transition state locating. Optimized geometries were obtained by minimizing the forces on the atoms below 0.02 eV $Å^{-1}$. The transition state was first isolated using the climbing image nudged elastic band (CI-NEB) method and then refined using the dimer method to until force is below 0.02 eV $Å^{-1}$. The resulting transition state was finally confirmed by the normal mode frequency analysis, showing only one imaginary mode. The PBE type of PAW potential was displayed in Supplementary Figure 19. After that, the newly developed GW potential in potpaw_PBE.5.2, with the valence electronic configuration of Ti ($3s^23p^63d^4$), O ($2s^22p^4$), Ru ($4s^24p^64d^8$), and C ($2s^22p^2$) was adopted for the further optimization of adsorption geometries and transition states.

Supplementary Figures

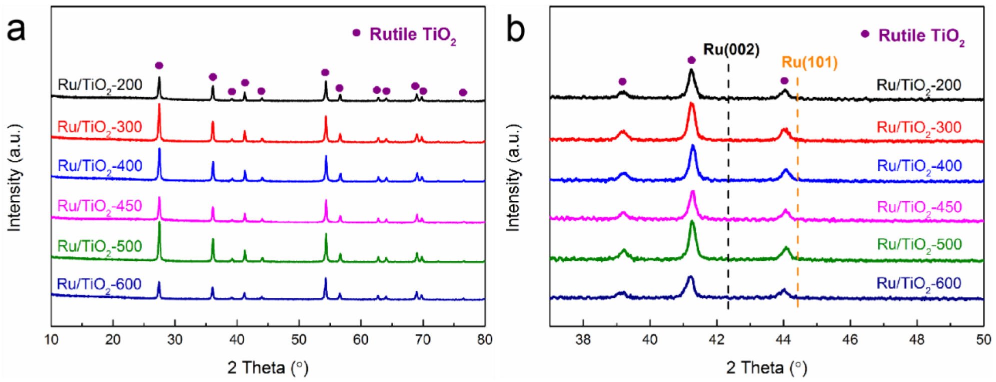

Supplementary Figure 1. (a) XRD patterns of the Ru/TiO_2 catalysts pretreated at different temperatures (Ru/TiO_2-*x* samples) and (b) partially enlarged details.

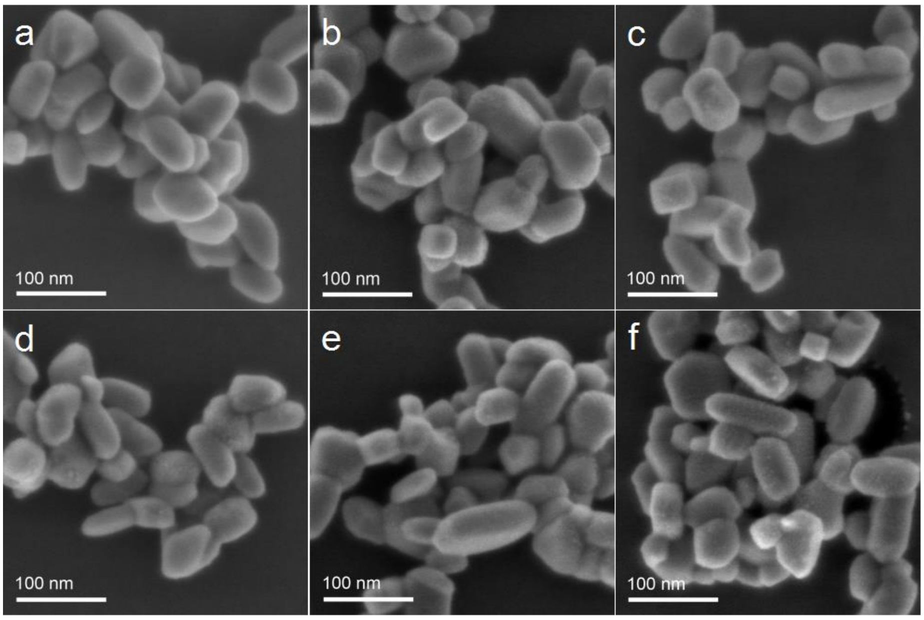

Supplementary Figure 2. HRSEM images of the Ru/TiO_2-*x* catalysts: (a) Ru/TiO_2-200; (b) Ru/TiO_2-300; (c) Ru/TiO_2-400; (d) Ru/TiO_2-450; (e) Ru/TiO_2-500; (f) Ru/TiO_2-600.

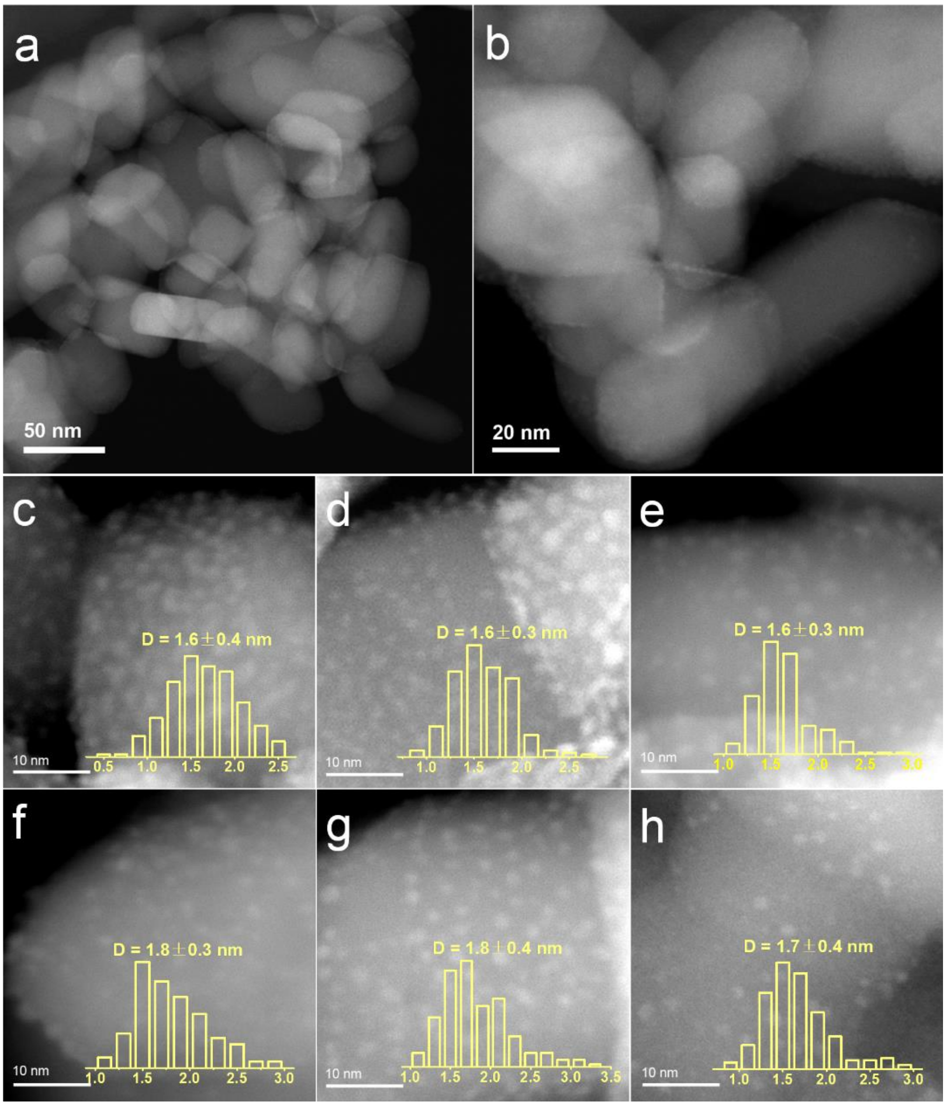

Supplementary Figure 3. (a, b) Low resolution HAADF-STEM images of the fresh Ru/TiO_2 catalyst. (c–h) HAADF-STEM images of the Ru/TiO_2 catalysts pretreated at different temperatures (Ru/TiO_2-*x* samples) with the metal size distribution. (c) Ru/TiO_2-200; (d) Ru/TiO_2-300; (e) Ru/TiO_2-400; (f) Ru/TiO_2-450; (g) Ru/TiO_2-500; (h) Ru/TiO_2-600.

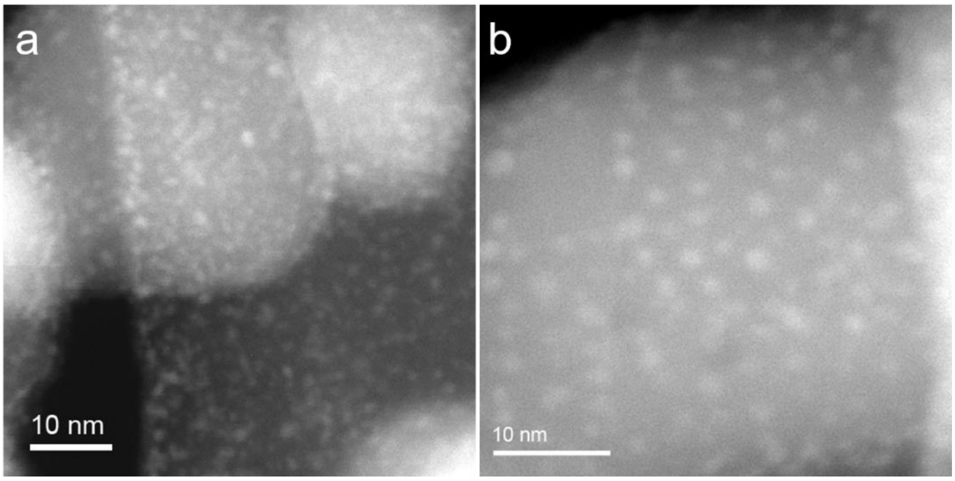

Supplementary Figure 4. HAADF-STEM images of Ru/TiO_2 samples upon different steps. (a) Fresh $RuCl_3$/TiO_2 catalyst after impregnation and drying overnight. (b) The obtained Ru/TiO_2-500 catalyst after thermal treatment in air at 300 °C followed by reduction in H_2 at 500 °C.

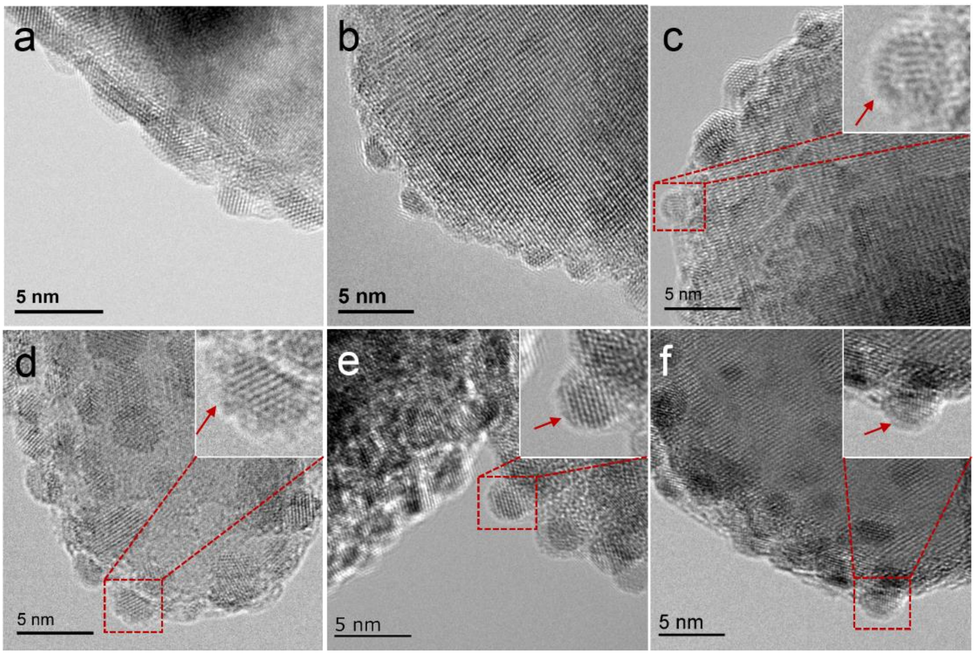

Supplementary Figure 5. HRTEM images of the Ru/TiO_2 catalysts pretreated at different temperatures (Ru/TiO_2-*x* samples). (a) Ru/TiO_2-200; (b) Ru/TiO_2-300; (c) Ru/TiO_2-400; (d) Ru/TiO_2-450; (e) Ru/TiO_2-500; (f) Ru/TiO_2-600.

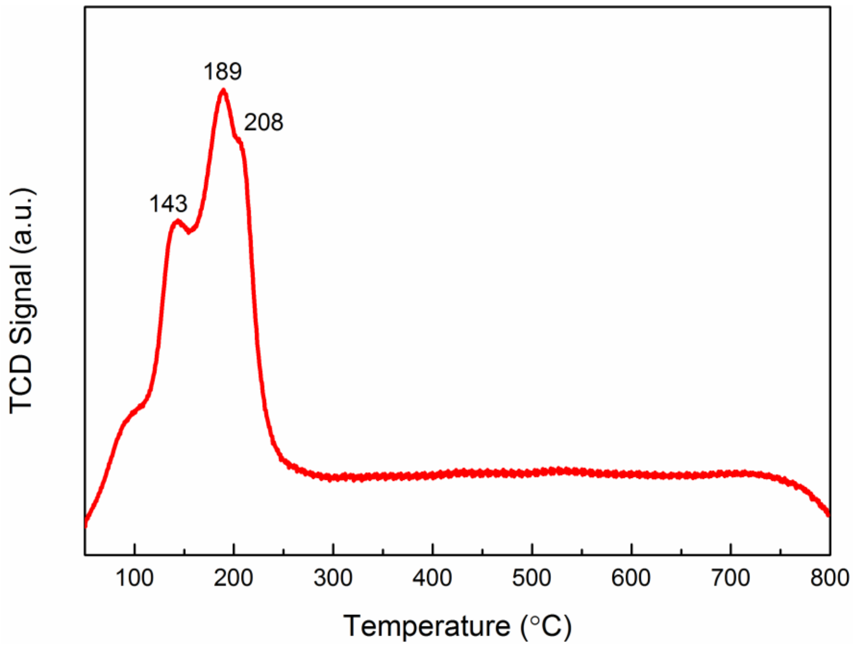

Supplementary Figure 6. H_2-TPR profile obtained from the fresh Ru/TiO_2 catalyst.

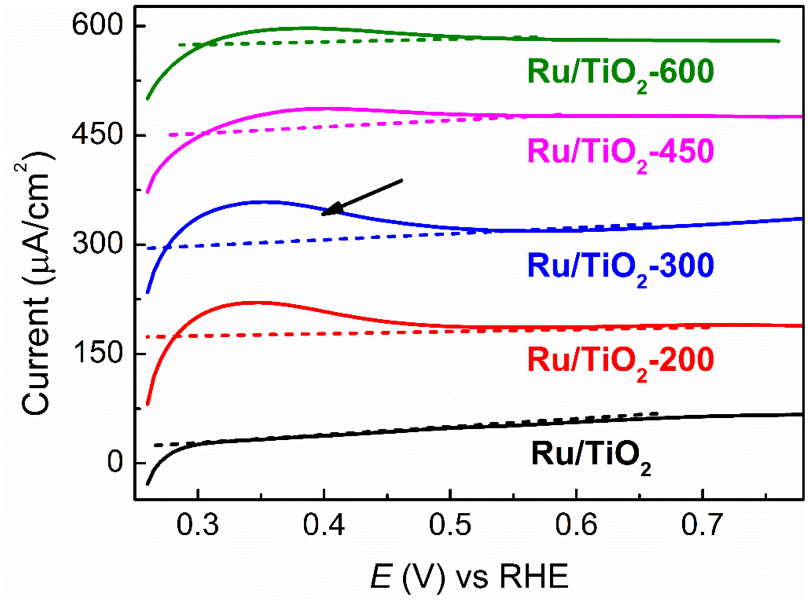

Supplementary Figure 7. Underpotential deposition of copper (Cu upd) on the fresh Ru/TiO_2 catalyst and the Ru/TiO_2-*x* catalysts.

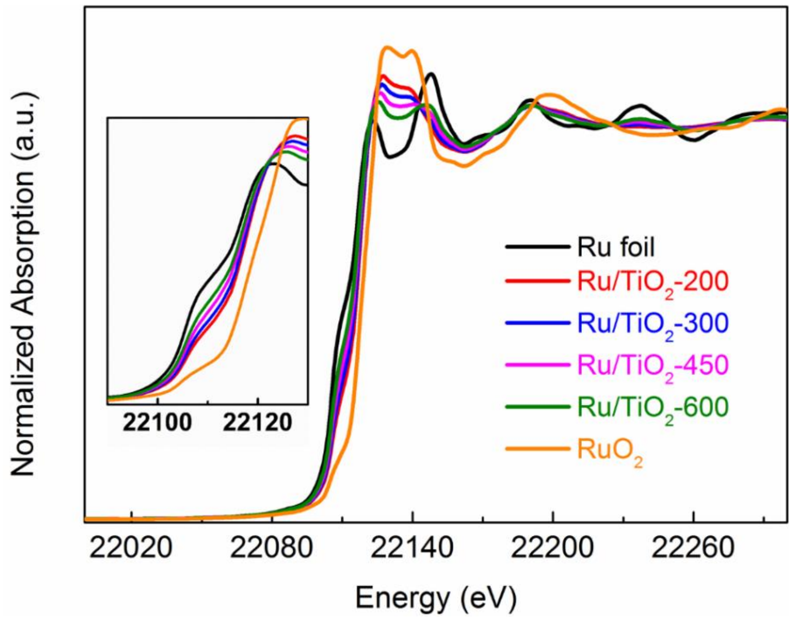

Supplementary Figure 8. Normalized XANES spectra at the Ru *K*-edge for the Ru/TiO_2-*x* catalysts.

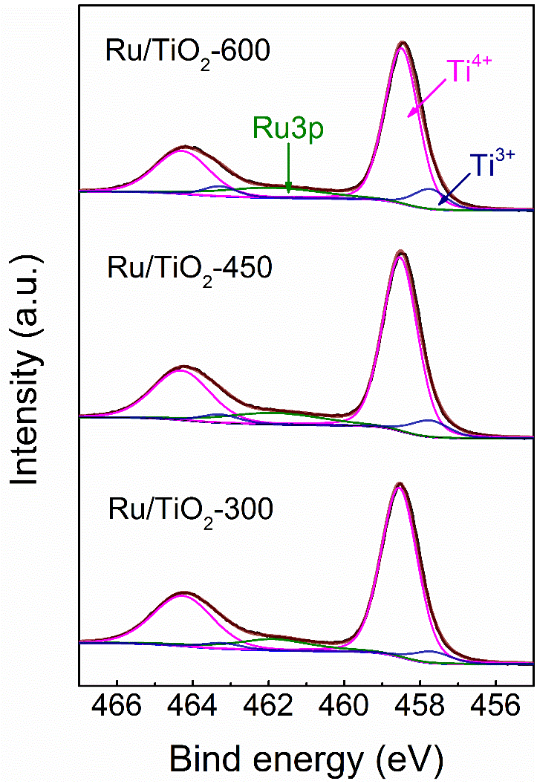

Supplementary Figure 9. Ti 2*p* and Ru 3$p_{3/2}$ XP spectra of the Ru/TiO_2-*x* catalysts.

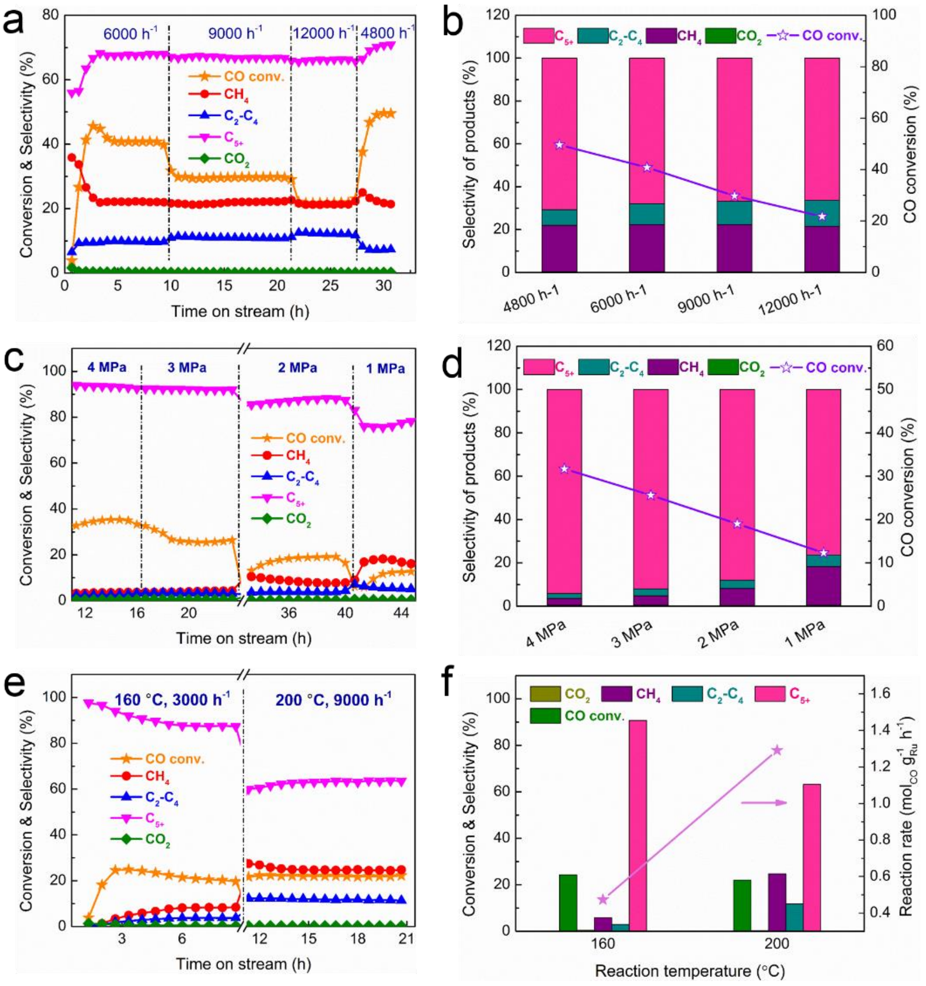

Supplementary Figure 10. (a, b) The effect of the space velocity over the Ru/TiO_2-450 catalyst (reaction conditions: 200 °C, 2 MPa, H_2/CO/Ar = 64/32/4). (c, d) The effect of reaction pressure over the Ru/TiO_2-450 catalyst (reaction conditions: 160 °C, space velocity = 3000 mL h^{-1} g_{cat}^{-1}, H_2/CO/Ar = 64/32/4). (e, f) The effect of reaction temperature over the Ru/TiO_2-450 catalyst (reaction conditions: 2 MPa, space velocity = 3000 mL h^{-1} g_{cat}^{-1} for 160 °C and 9000 mL h^{-1} g_{cat}^{-1} for 200 °C, H_2/CO/Ar = 64/32/4).

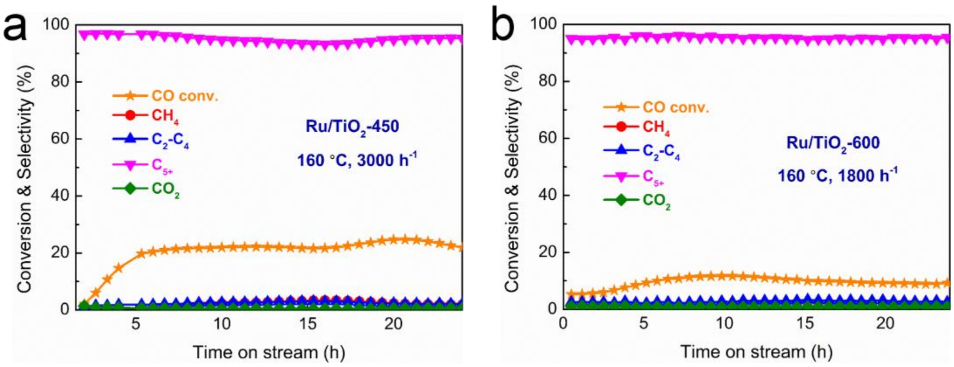

Supplementary Figure 11. Evolution of catalytic performance versus time over the (a) Ru/TiO_2-450, (b) Ru/TiO_2-600 catalyst.

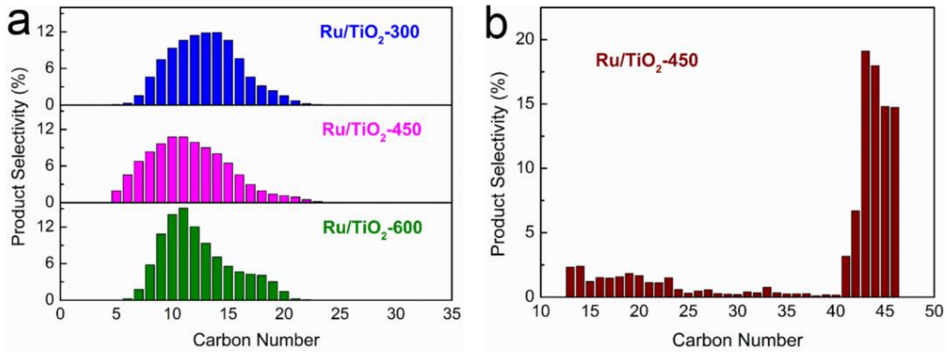

Supplementary Figure 12. The carbon number distribution (a) Liquid hydrocarbons; (b) Solid wax.

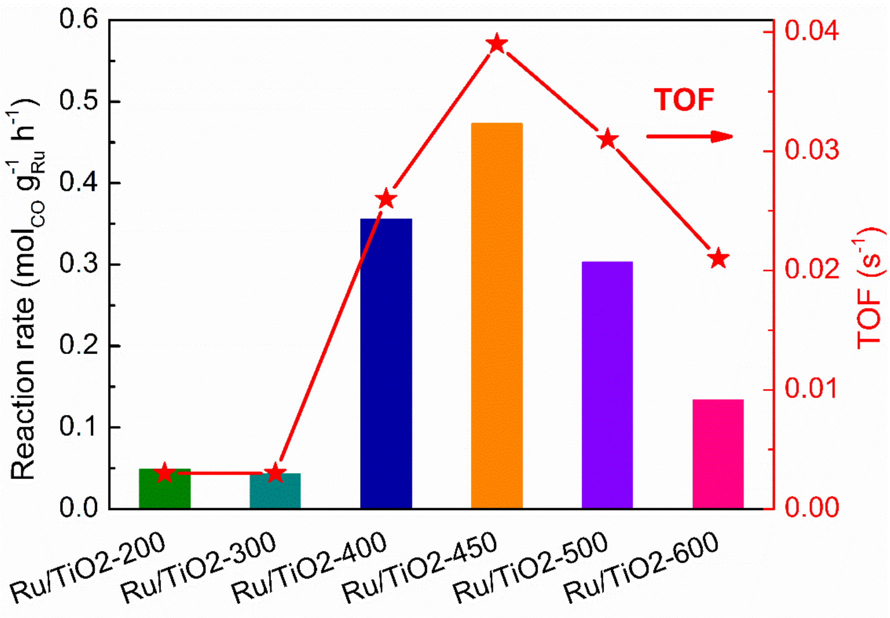

Supplementary Figure 13. Reaction rates and TOF values for the Ru/TiO_2-*x* catalysts.

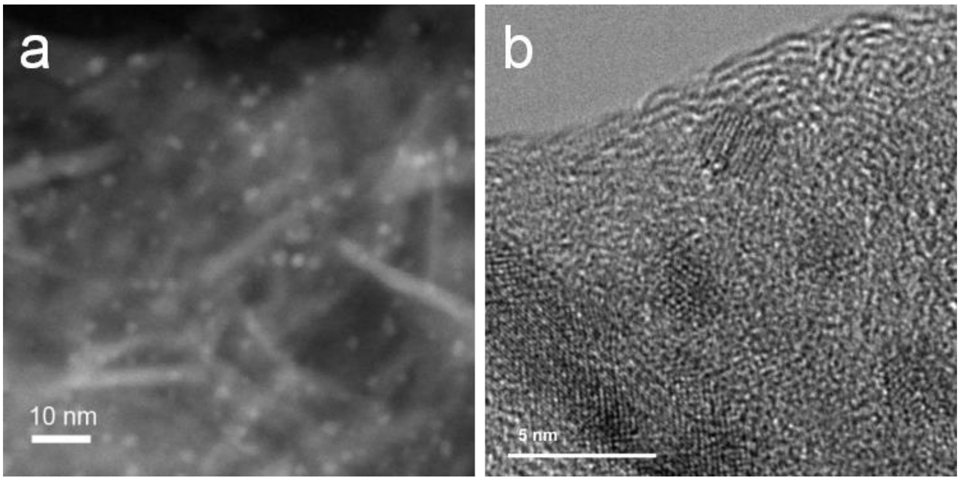

Supplementary Figure 14. (a) HAADF-STEM and (b) HRTEM images of the spent Ru/Al_2O_3-450 catalyst.

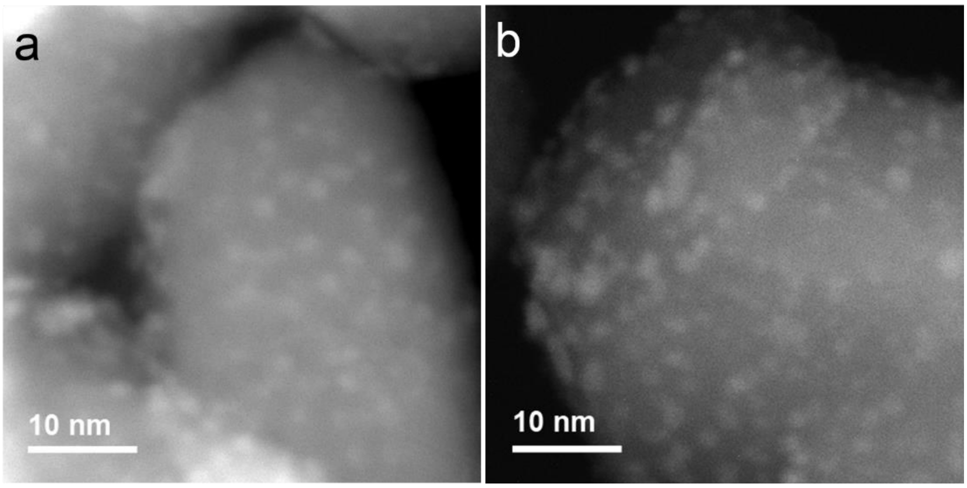

Supplementary Figure 15. HAADF-STEM images of (a) the fresh Ru/TiO_2-450 and (b) Ru/TiO_2-450-spent catalyst.

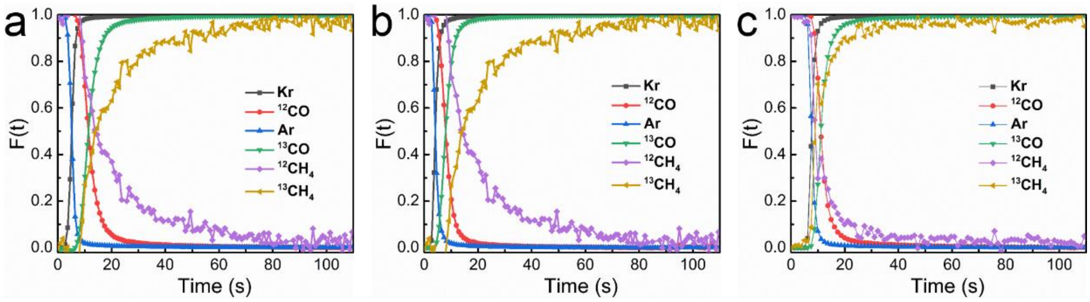

Supplementary Figure 16. Normalized transient curves for the Ru/TiO_2-*x* catalysts after a switch from $^{12}CO/H_2/Ar$ to $^{13}CO/H_2/Kr$ (SSITKA performing condition: 200 °C, 0.185 MPa, H_2/CO = 10). (a) Ru/TiO_2-300; (b) Ru/TiO_2-450; (c) Ru/TiO_2-600. F(t) is the normalized transient response.

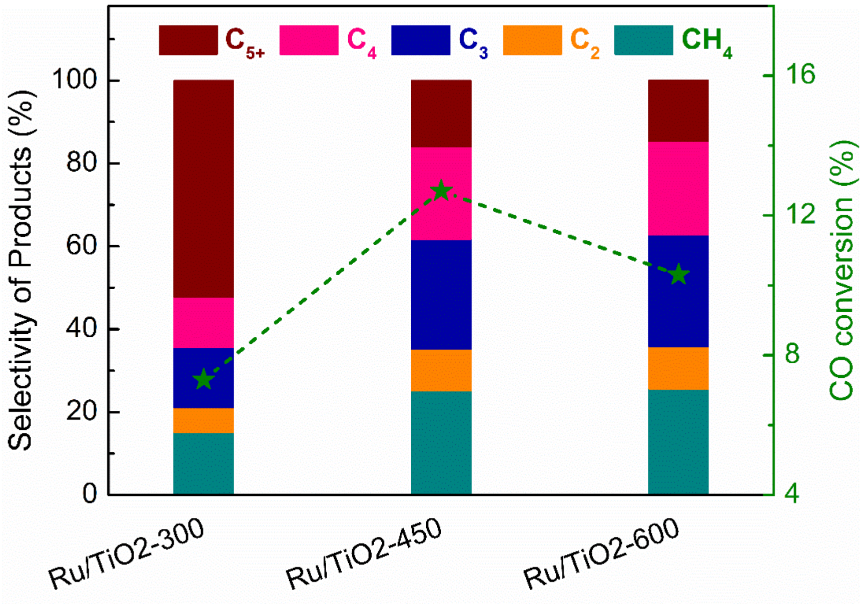

Supplementary Figure 17. Catalytic Performance of the Ru/TiO_2-*x* catalysts in SSITKA experiments. Performing condition: 200 °C, 0.185 MPa, H_2/CO = 10.

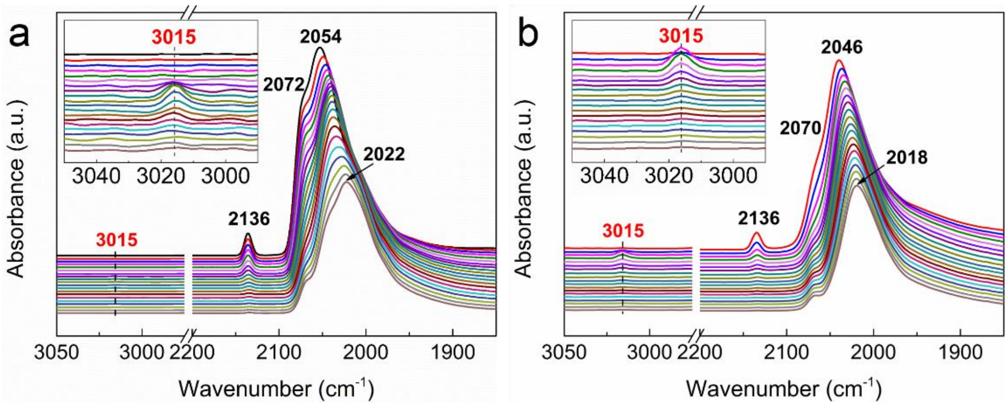

Supplementary Figure 18. Evolution of the CO_{ad} species during H_2 flow at 160 °C as determined using *in situ* DRIFT spectra, over the Ru/TiO_2-300 and Ru/TiO_2-600 catalyst, with partially enlarged details in their panels.

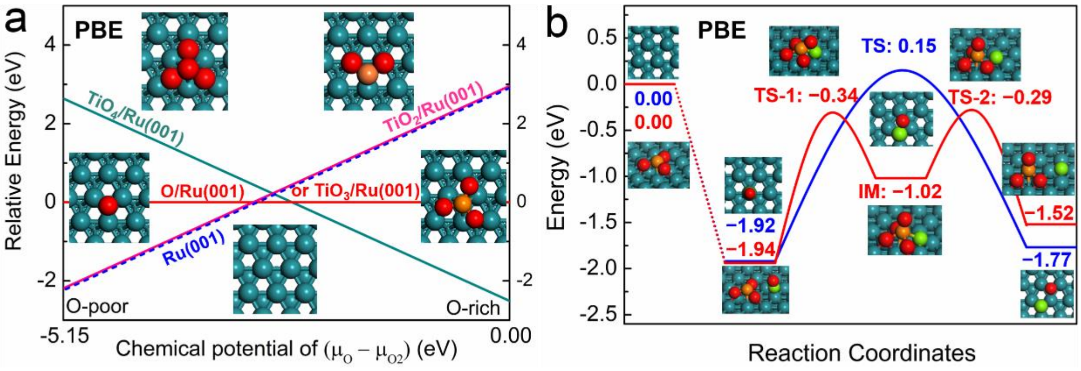

Supplementary Figure 19. (a) Thermodynamic stability of different TiO_x/Ru(001) and O/Ru(001) under a variation of the chemical potential of O, with referring to TiO_3/Ru(001) and Ru(001), respectively, with the atomic configuration in insets. (b) A possible catalytic mechanisms of CO activation at PBE level calculations.

Supplementary Tables

Supplementary Table 1. BET surface areas and pore volumes of the Ru/TiO_2-*x* catalysts.

Sample	S_{BET} (m^2 g^{-1})	V_{pore} (cm^3 g^{-1})
Ru/TiO_2-200	36	0.060
Ru/TiO_2-300	33	0.054
Ru/TiO_2-400	39	0.069
Ru/TiO_2-450	37	0.075
Ru/TiO_2-500	37	0.066
Ru/TiO_2-600	37	0.065

Supplementary Table 2. The crystal parameters of rutile-type RuO_2 and TiO_2.

Oxide	Crystal form	M–O bond distance (nm)	Lattice spacing (nm)	
			a, b axis	c axis
RuO_2	rutile	0.1941	0.4497	0.3105
TiO_2	rutile	0.1980	0.4594	0.2958

Supplementary Table 3. The results obtained by underpotential deposition of copper (Cu upd).

Catalyst	A (cm^2)	S_{sp}（$m^2\ g^{-1}$）	Dispersion (%)
Ru/TiO_2	0	–	–
Ru/TiO_2-200	0.300	–	–
Ru/TiO_2-300	0.405	30.7	8.1
Ru/TiO_2-450	0.202	15.3	4.0
Ru/TiO_2-600	0.189	14.2	3.8

Supplementary Table 4. EXAFS fitting results for the Ru/TiO_2-*x* catalysts.[a]

Sample	Shell	CN	R (Å)	$\sigma^2 \times 10^2$ (Å^2)	ΔE_0 (eV)	R factor
Ru foil	Ru–Ru	12	2.68	0.35	4.3	0.013
RuO_2	Ru–O	6	1.97	0.26	4.4	0.005
Ru/TiO_2-200	Ru–Ru	2.2	2.67	0.77	5.3	0.005
	Ru–O	4.0	1.98	0.71		
Ru/TiO_2-300	Ru–Ru	3.2	2.67	0.71	5.8	0.008
	Ru–O	3.8	1.98	0.80		
Ru/TiO_2-450	Ru–Ru	4.2	2.67	0.58	3.2	0.007
	Ru–O	3.3	1.98	0.90		
Ru/TiO_2-600	Ru–Ru	5.3	2.66	0.53	3.4	0.010
	Ru–O	2.4	1.98	0.71		

[a] CN, the coordination number for the absorber-backscatterer pair. R, the average absorber-backscatterer distance. σ^2, the Debye-Waller factor. ΔE_0, the inner potential correction. The accuracies of the above parameters were estimated as: N, ±20%; R, ±1%; σ^2, ±20%; ΔE_0, ±20%. The data range used for data fitting in *k*-space (Δk) and *R*-space (ΔR) were 3.0–14.1 Å^{-1} and 1.0–3.1 Å, respectively.

Supplementary Table 5. Quantified XPS data for surface Ti and Ru/Ti ratio on the Ru/TiO_2-*x* catalysts.

Sample	Ti^{4+}			Ti^{3+}			Ru/Ti
	B.E. (eV)		Content	B.E. (eV)		Content	ratio
	$2p_{1/2}$	$2p_{3/2}$	(%)	$2p_{1/2}$	$2p_{3/2}$	(%)	(%)
Ru/TiO_2-300	464.3	458.6	93.3	463.2	457.7	6.7	12.9
Ru/TiO_2-450	464.3	458.5	90.5	463.3	457.8	9.5	11.6
Ru/TiO_2-600	464.3	458.5	88.4	463.3	457.8	11.6	10.7

Supplementary Table 6. TOF values calculated by CO chemisorption for the Ru/TiO_2-*x* catalysts.

Sample	CO uptake (μmol g^{-1})	Ru dispersion (%)	Reaction rate (mol_{CO} g_{Ru}^{-1} h^{-1})	TOF (s^{-1})
Ru/TiO_2-200	102.6	47.2	0.049	0.003
Ru/TiO_2-300	94.2	43.3	0.043	0.003
Ru/TiO_2-400	84.7	38.9	0.356	0.026
Ru/TiO_2-450	74.0	34.0	0.473	0.039
Ru/TiO_2-500	59.8	27.5	0.303	0.031
Ru/TiO_2-600	38.0	17.5	0.134	0.021

Supplementary Table 7. Comparison of the catalytic behaviors of Ru-based catalysts.

Catalyst	Ru particle size (nm)	Temp. (°C)	Reaction rate (mol_{CO} g_{Ru} h^{-1})	TOF (s^{-1})	Ref.
Ru/TiO_2-450	1.8 ± 0.4	200	1.697	0.140	This work
		160	0.473	0.039	
Ru/TiO_2 (R)	-	250	0.547	-	4
Ru/TiO_2 (A)	-	250	0.403	-	
Ru/TiO_2 (R+A)	-	250	0.482	-	
Ru/Al_2O_3	-	250	0.277	-	
Ru/Al_2O_3-PHR	2.5 ± 0.5	150	0.129	0.006	5
Ru/Al_2O_3-10Cl	2.2 ± 0.5	250	0.333	0.03	6
2% Ru/TiO_2	-	275	-	0.390	7
5% Ru/Al_2O_3	-	275	-	0.266	
5% Ru/SiO_2	-	275	-	0.090	
4% Ru/Carbolac	-	275	-	0.018	
Ru/TiO_2	1.7 ± 0.2	230	0.066	-	8
Ru/CeO_2	1.6 ± 0.2	230	0.060	-	
Ru/C	1.5 ± 0.2	230	0.077	-	
Ru@Si/Al-10	11.6	270	0.121	-	9
Ru@SiAl-30	13.9	270	0.303	-	

Ru@SiAl-50	16.1	270	0.404	-	
Ru/meso-ZSM-5	6.6	260	0.507	0.068	10
Ru/meso-beta	7.2	260	0.531	0.071	11
Ru/HB-S	2.9	260	-	0.129	12
Ru/CNT	6.3	260	0.583	0.193	13
Ru@MHCS	3.2	250	0.342	0.023	14
Ru@HCS	5.5	250	0.162	0.021	

Supplementary Table 8. Catalytic performances of the Ru/Al_2O_3-450 catalyst.

Reaction temp. (°C)	CO conv. (%)	Reaction rate (mol_{CO} g_{Ru}^{-1} h^{-1})	Selectivity (%)			
			CO_2	CH_4	C_{2-4}	C_{5+}
160	1.3	0.013	4.8	4.7	11.6	78.9
180	3.3	0.032	29.5	5.4	9.3	55.8
200	11.3	0.112	41.5	4.2	6.9	47.4
220	31.3	0.310	18.7	4.4	6.5	70.4

Reaction conditions: 2 MPa, space velocity = 1800 mL h^{-1} g_{cat}^{-1}, H_2/CO/Ar = 64/32/4.

Supplementary Table 9. Relative energy of successive reduction steps under O-rich and O-poor condition.

Reduction step	O-rich (eV)	O-poor (eV)
$TiO_4 \rightarrow TiO_3 + O$	2.66	−2.49
$TiO_3 \rightarrow TiO_2 + O$	2.94	−2.21
O/Ru(001)→Ru(001)+ O	2.87	−2.28

Supplementary Notes

Supplementary Note 1. As shown in Supplementary Figure 1, the XRD patterns of the Ru/TiO_2-*x* catalysts do not show the characteristic peak for Ru^0, indicating that the Ru particles were highly dispersed on the rutile TiO_2 with their sizes below the limit of detection.

Supplementary Note 2. The H_2-TPR profile of the fresh Ru/TiO_2 catalyst displays three main peaks, ascribed to the reduction of RuO_2 species with different interfacial interactions with the TiO_2 (Supplementary Figure 6). The peaks (at 189 and 208 °C) show a higher temperatures than those in previous reports[15, 16, 17], demonstrating the presence of strong interactions between the RuO_2 and rutile TiO_2 due to the lattice match of oxides. Consequently, the Ru/TiO_2 catalyst was stable so as to avoid particle growth during reduction. A less intense, broad peak also appears between 300 and 800 °C, attributed to reduction of the TiO_2 support due to H spillover from the Ru to the TiO_2.

Supplementary Note 3. The evolution of the surface metallic Ru exposure with the increase of pre-reduction temperatures was also determined by Cu upd experiments. This technique has been proven to be an effective method for quantifying the specific metal surface area, and the integral area of current for the reduction deposition of copper is proportionate to the exposed metal surface[18]. Supplementary Figure 7 and

Supplementary Table 3 show the results of Cu upd for different Ru/TiO_2-*x* samples. Obviously, no metallic Ru was detected for the fresh Ru/TiO_2 sample due to the only presence of RuO_2 before reduction. In contrast, a great amount of metallic Ru was distinguished on the Ru/TiO_2-200 sample, which was attributed to the incipient reduction of Ru/TiO_2 at 200 °C as confirmed by H_2 temperature-programmed reduction (H_2-TPR) in Supplementary Figure 6. The exposure of metallic Ru reached a maximum on the Ru/TiO_2-300 sample, and a remarkable decline was observed with further increasing pretreatment temperature. This can be explained by a gradual encapsulation of the Ru NPs by TiO_x overlayer as increasing the reduction temperature from 300 to 600 °C, which was in good agreement with the TEM observations.

However, Cu upd shows a much lower dispersion than that of CO chemisorption. It might be caused by the Ru^{n+} sites at the Ru-TiO_2 interface, which are unavailable for the method of Cu underpotential deposition, but it can be contained in the CO chemisorption.

Supplementary Note 4. As shown in Supplementary Figure 8, the edge energies of the Ru/TiO_2-*x* catalysts are located between those of the Ru foil and RuO_2 standard. With increasing in the reduction temperature from 200 to 600 °C, the catalysts exhibited a shift towards lower energies closer to the Ru foil, indicative an improving degree of reduction of the Ru species with increasing reduction temperature. Despite its relatively high degree of reduction, the Ru/TiO_2-600 sample was still incompletely

reduced due to the strong interaction between the RuO_2 and TiO_2 at the interface, which is in good agreement with the H_2-TPR results.

Supplementary Note 5. XPS was also employed to investigate the chemical state of the TiO_2. The Ti 2*p* XP spectra in Supplementary Figure 9 and Supplementary Table 5 demonstrate that increasing the reduction temperature from 300 to 600 °C increased the Ti^{3+} concentration from 6.7% to 11.6%. These data are in good agreement with the XANES results for TiO_2.

Supplementary Note 6. The liquid and solid products (C_{5+}) were analyzed offline using an Agilent 7890 gas chromatograph equipped with an HP-5 capillary column connected to a flame ionization detector (FID). The liquid hydrocarbons were dissolved in ethanol, while the solid wax was dissolved in dodecane. The C_{5+} products consist of main normal paraffins and a fraction of alkenes. The relative content of each product was detected by the normalization method of peak area. As shown in Supplementary Figure 12, the carbon number distribution of liquid hydrocarbons mainly concentrates in C_5–C_{20}, while that of solid wax consists a great mount of C_{40}–C_{46} hydrocarbons.

Supplementary Note 7. The TOF values were calculated by using the Ru dispersion determined from CO chemisorption, and the results were shown in Supplementary Figure 13 and Supplementary Table 6. The variation in TOF value exhibits a

volcano-type trend with increasing the pretreatment temperature from 200 to 600 °C.

Supplementary Note 8. HAADF-STEM image of the spent Ru/TiO_2-450 catalyst suggests that the size of Ru can keep constant after testing (Supplementary Figure 15). This was also benefited from the SMSI in the Ru/TiO_2-450 catalyst, which greatly prohibits the size aggregation of Ru during FTS process.

Supplementary References

1. Reuter K, Scheffler M. Composition, structure, and stability of RuO_2(110) as a function of oxygen pressure. *Phys Rev B* **65**, 035406 (2001).
2. Lide DR. CRC Handbook of Chemistry and Physics, 88th Edition. *Taylor & Francis Group, Boca Raton*, (2007).
3. Lejaeghere K*, et al.* Reproducibility in density functional theory calculations of solids. *Science* **351**, aad3000 (2016).
4. Kikuchi E, Matsumoto M, Takahashi T, Machino A, Morita Y. Fischer-Tropsch synthesis over titania-supported ruthenium catalysts. *Appl Catal* **10**, 251–260 (1984).
5. Lian C, Yu Y, Zhang K, Gao A, Wang Y. Highly efficient Fischer–Tropsch synthesis over an alumina-supported ruthenium catalyst. *Catal Sci Technol* **8**, 1528–1534 (2018).
6. González-Carballo JM, Pérez-Alonso FJ, García-García FJ, Ojeda M, Fierro JLG, Rojas S. In-situ study of the promotional effect of chlorine on the Fischer–Tropsch synthesis with Ru/Al_2O_3. *J Catal* **332**, 177–186 (2015).
7. Vannice MA, Garten RL. The influence of the support on the catalytic behavior of ruthenium in CO/H_2 synthesis reactions. *J Catal* **63**, 255–260 (1980).
8. Koh T, Koo HM, Yu T, Lim B, Bae JW. Roles of Ruthenium-support interactions of size-controlled ruthenium nanoparticles for the product distribution of Fischer-Tropsch synthesis. *ACS Catal* **4**, 1054–1060 (2014).

9. Hwang J, *et al.* Direct confinement of Ru nanoparticles inside nanochannels of large pore mesoporous aluminosilicate for Fischer–Tropsch synthesis. *J Mater Chem A* **3**, 23725–23731 (2015).

10. Kang J, *et al.* Mesoporous zeolite-supported ruthenium nanoparticles as highly selective Fischer-Tropsch catalysts for the production of C_5-C_{11} isoparaffins. *Angew Chem Int Ed* **50**, 5200–5203 (2011).

11. Cheng K, *et al.* Mesoporous beta zeolite-supported ruthenium nanoparticles for selective conversion of synthesis gas to C_5–C_{11} isoparaffins. *ACS Catal* **2**, 441–449 (2012).

12. Sun J, *et al.* Highly-dispersed metallic Ru nanoparticles sputtered on H-beta zeolite for directly converting syngas to middle isoparaffins. *ACS Catal* **4**, 1–8 (2014).

13. Kang J, Zhang S, Zhang Q, Wang Y. Ruthenium nanoparticles supported on carbon nanotubes as efficient catalysts for selective conversion of synthesis gas to diesel fuel. *Angew Chem Int Ed* **48**, 2565–2568 (2009).

14. Phaahlamohlaka TN, Kumi DO, Dlamini MW, Jewell LL, Coville NJ. Ruthenium nanoparticles encapsulated inside porous hollow carbon spheres: a novel catalyst for Fischer–Tropsch synthesis. *Catal Today* **275**, 76–83 (2016).

15. Lin Q, Liu XY, Jiang Y, Wang Y, Huang Y, Zhang T. Crystal phase effects on the structure and performance of ruthenium nanoparticles for CO_2 hydrogenation. *Catal Sci Technol* **4**, 2058–2063 (2014).

16. Carballo JMG, *et al.* Insights into the deactivation and reactivation of Ru/TiO_2

during Fischer–Tropsch synthesis. *Catalysis Today* **214**, 2-11 (2013).

17. Omotoso T, Boonyasuwat S, Crossley SP. Understanding the Role of TiO_2 Crystal Structure on the Enhanced Activity and Stability of Ru/TiO_2 Catalysts for the Conversion of Lignin-Derived Oxygenates. *Green Chem* **16**, 645–652 (2014).

18. Green CL, Kucernak A. Determination of the platinum and ruthenium surface areas in platinum-ruthenium alloy electrocatalysts by underpotential deposition of copper. I. unsupported catalysts. *J Phys Chem B* **106**, 1036–1047 (2002).
